# IL-6 produced by prostate epithelial cells stimulated with *Trichomonas vaginalis* promotes proliferation of prostate cancer cells by inducing M2 polarization of THP-1-derived macrophages

**DOI:** 10.1371/journal.pntd.0008126

**Published:** 2020-03-20

**Authors:** Ik-Hwan Han, Hyun-Ouk Song, Jae-Sook Ryu

**Affiliations:** 1 Department of Environmental Biology and Medical Parasitology, Hanyang University College of Medicine, Seoul, Korea; 2 Department of Parasitology, School of Medicine, Catholic University of Daegu, Daegu, Korea; George Washington University, UNITED STATES

## Abstract

*Trichomonas vaginalis* (*Tv*), a protozoan parasite causing sexually-transmitted disease, has been detected in tissue of prostatitis, benign prostatic hyperplasia (BPH) and prostate cancer (PCa). IL-6, a mediator of chronic inflammation, induces the progression of prostate cancer, and influences the polarization of M2 macrophages, which are the main tumor-associated macrophages. We investigated whether IL-6 produced by human prostate epithelial cells stimulated with *Tv* induces the M2 polarization of THP-1-derived macrophages, which in turn promotes the progression of PCa. Conditioned medium was prepared from *Tv-*infected (TCM) and uninfected (CM) prostate epithelial cells (RWPE-1). Thereafter conditioned medium was prepared from macrophages after incubation with CM (M-CM) or TCM (M-TCM). RWPE-1 cells infected with *Tv* produced IL-6 and chemokines such as CCL2 and CXCL8. When human macrophages were treated with conditioned medium of RWPE-1 cells co-cultured with *Tv* (TCM), they became polarized to M2-like macrophages as indicated by the production of IL-10 and TGF-β, and the expression of CD36 and arginase-1, which are M2 macrophage markers. Moreover, proliferation of the M2-like macrophages was also increased by TCM. Blockade of IL-6 signaling with IL-6 receptor antibody and JAK inhibitor (Ruxolitinib) inhibited M2 polarization of THP-1-derived macrophages and proliferation of the macrophages. To assess the effect of crosstalk between macrophages and prostate epithelial cells inflamed by *Tv* infection on the growth of prostate cancer (PCa) cells, PC3, DU145 and LNCaP cells were treated with conditioned medium from THP-1-derived macrophages stimulated with TCM (M-TCM). Proliferation and migration of the PCa cells were significantly increased by the M-TCM. Our findings suggest that IL-6 produced in response to *Tv* infection of the prostate has an important effect on the tumor microenvironment by promoting progression of PCa cells following induction of M2 macrophage polarization.

## Author summary

In male, *T*. *vaginalis* infection have been proposed to involve in several prostate diseases such as prostatitis, benign prostatic hyperplasia and prostate cancer. However, studies for these mechanisms have been rare. We have previously reported that *T*. *vaginalis* induce the production of inflammatory cytokines in prostate cells. Among these cytokines, IL-6 have been reported to play an important role in M2 macrophage polarization, which lead to formation of tumor microenvironment in various cancers. Here we show that IL-6 produced by *T*. *vaginalis* infection in prostate epithelial cells induces M2 polarization of macrophages and these macrophages promote proliferation of prostate cancer cells. These findings suggest that *T*. *vaginalis* indirectly induces progression of prostate cancer by creating a tumor microenvironment through an inflammatory response.

## Introduction

Trichomoniasis is the most common curable sexually transmitted disease (STD); it is caused by infection with the protozoan parasite *Trichomonas vaginalis* (*T*. *vaginalis*). In men, trichomoniasis is associated with urethritis and prostatitis [1, 2]. Some studies have suggested that *T*. *vaginalis* is a factor causing chronic prostatitis and benign prostatic hyperplasia (BPH), as well as increasing the risk of prostate cancer [3–5]. In particular, as evidence of the association between *T*. *vaginalis* infection and prostate cancer, macrophage migration inhibitory factor (MIF) secreted from *T*. *vaginalis* has been reported to induce proliferation of prostate cancer cells [6]. Recently, prostate cancer patients showed higher seropositivity against *T*. *vaginalis* than normal men in Korea [7]. On the other hand, other authors have reported clinical evidence that there is no association between *T*. *vaginalis* infection and prostate cancer [8, 9]. The association between *T*. *vaginalis* infection and prostate cancer is still controversial. However, Simons et al. reported that the chronic inflammation of prostate by bacterial infections induce the progression of prostate cancer [10]. *T*. *vaginalis* infection in men is asymptomatic or have only mild symptoms [11, 12]. Therefore, persistent infection with *T*. *vaginalis* has been hypothesized to cause chronic inflammation [5].

Inflammation has been implicated as a significant contributor to the initiation and progression of a wide range of malignancies, including prostate cancer [13]. An estimated 20% of all cancers are now thought to be attributable to chronic inflammatory conditions caused by infectious agents, chronic noninfectious inflammatory diseases and other environmental factors [14]. Histologic studies have found signs of immune infiltration in 80–90% of prostate cancer specimens and high-grade disease was associated with increased inflammation [15]. Many cells of the innate immune system, such as macrophages, dendritic cells and mast cells infiltrate inflamed tissues and have been implicated in the development and progression of cancer by contributing to the tumor microenvironment [16]. In previous studies, we showed that the conditioned medium containing inflammatory mediators of prostate cells infected by *T*. *vaginalis* attracted immune cells such as monocytes, neutrophils and mast cells [17–19].

Macrophages play a key role in chronic inflammation, and are the most abundant immune cells in the tumor microenvironment, the so-called tumor-associated macrophages (TAMs) [20]. Recently, immunological studies have identified two distinct functional macrophage phenotypes: the ‘classical’ activated (M1) macrophage and the ‘alternatively’ activated (M2) macrophage [21]. M1 macrophages can destroy tumor cells by producing inflammatory cytokines such as tumor necrosis factor (TNF)-α, IL-12, CXCL10 (IP10), CCL2 and nitric oxide (NO). In contrast, M2 macrophages promote tissue repair and angiogenesis, and favor tumor progression by producing anti-inflammatory cytokines such as interleukin (IL)-4, IL-10, IL-13 and tumor growth factor (TGF)-β, and high level expression of arginase-1, and can be identified by the expression of CD36, CD163, CD206 and CD301 on their cell surface [22, 23]. TAMs are mainly composed of M2-type macrophages and play crucial roles in the survival, proliferation and metastasis of cancer cells [24].

M1 macrophages are induced by Th1 (pro-inflammatory) cytokines such as TNF-α, interferon (INF)-γ, and microbial products such as lipopolysaccharide (LPS), whereas M2 macrophages are induced by Th2 (anti-inflammatory) cytokines, such as IL-4, IL-13, and partially by IL-10 [25]. Interestingly, IL-6, which is a classical proinflammatory cytokine upregulated during several kinds of inflammatory process, has been reported to induce polarization to the M2-phenotype [26, 27]. Macrophages accumulate in sites of inflammation, but in Th2 inflammation M2 macrophages induce the proliferation of local macrophages rather than recruiting them from circulating monocytes [27, 28].

The aim of the present study was to investigate the polarization of M2 macrophages in response to inflammation of prostate epithelial cells exposed to *T*. *vaginalis*, and progression of prostate cancers as a result of interaction of the cancer cell with products of the polarized macrophages. Our findings suggest that prostate epithelial cells produce IL-6 when inflamed by *T*. *vaginalis* infection, this polarizes macrophages to the M2 type, and crosstalk with these differentiated (M2) macrophages promotes the proliferation and migration of prostate cancer cells.

## Materials and methods

### Ethics statement

All animal experimental protocols have been reviewed and approved by the Institutional Animal Care and Use Committee (IACUC) of Hanyang University under protocol number 2017-0151A. All animal experiments were handled in accordance with Korean Food and Drug Administration (KFDA) guidelines.

The human monocyte study protocols were reviewed and approved by the of Hanyang University institutional review board (IRB No. HYI-17-144-1). Peripheral blood was obtained from the Blood Center of Korean Red Cross as blood for research. Information about blood donors is private.

### Parasites and host cells

*Trichomonas vaginalis* isolate T016 isolate was kindly provided by Prof. John F. Alderete (School of Molecular Biosciences, College of Veterinary Medicine, Washington State University, WA, USA) [29] and grown in Diamond’s trypticase-yeast extract-maltose (TYM) medium supplemented with 10% heat-inactivated horse serum (Invitrogen, Carlsbad, CA, USA) at 37°C [30]. The human normal prostatic epithelial cell line (RWPE-1) was obtained from the American Type Culture Collection (ATCC; CRL-11609) and cultured in keratinocyte serum-free medium (K-SFM) supplemented with 25 μg/ml bovine pituitary extract (BPE), 5 ng/ml human recombinant epidermal growth factor (EGF), 100 U/ml penicillin, and 100 μg/ml streptomycin (Invitrogen, Carlsbad, CA, USA). The human monocytic leukemia cell line (THP-1) and human prostate cancer cell lines (PC3, DU145, and LNCaP) were purchased from the ATCC (Manassas, VA, USA). Cell lines were maintained in RPMI1640 (Hyclone, Logan, UT, USA) supplemented with 10% (v/v) fetal bovine serum (FBS) and 100 U/ml penicillin, and 100 μg/ml streptomycin at 37°C in 5% CO_2_.

### Macrophage differentiation and polarization

Human peripheral blood mononuclear cells (PBMCs) were isolated from normal donor buffy coat by gradient centrifugation using Histopaque-1077 (Sigma). The PBMCs were washed twice with PBS containing 2 mM EDTA and incubated in an erythrocyte lysis buffer (135 mM NH_4_Cl, 10 mM NaHCO_3_, 0.1 mM Na-EDTA, pH. 7.2) for 3 min at room temperature. Subsequently, PBMCs seeded at 5 × 10^6^ cells/ml on 24-well plates in RPMI1640 supplemented with 10% FBS and 50 ng/ml M-CSF (Peprotech, Rocky Hill, NJ, USA) for 2 hr to allow monocyte adhesion. Non-adherent cells, mostly T lymphocytes, were removed and the adherent monocytes (≥85% monocytes as determined by flow cytometric analysis after staining with anti-CD14 mAbs (BD biosciences)) were further incubated in RPMI1640 supplemented with 10% FBS and M-CSF (50 ng/ml) for 6 days. The medium was replaced every 3 days to obtain matured macrophages. The macrophages were polarized in M1 macrophages by incubation with 20 ng/mL of interferon (IFN)-γ (Peprotech) and with 100 ng/mL of lipopolysaccharide (LPS; Sigma-Aldrich) for 48 hr. Macrophage M2 polarization was obtained by incubation with 20 ng/mL of interleukin (IL)-4 (Peprotech) and 20 ng/mL of IL-13 (Peprotech) for 48 hr.

To obtain THP-1 macrophages, THP-1 cells were treated with 100 nM phorbol 12-myristate 13-acetate (PMA, Sigma-Aldrich, St. Louis, MO, USA) for 24 hr. M0 cells of differentiated non-polarized phenotype were obtained by treating monocytes with complete medium in the absence of PMA for 3 days. In order to generate polarized phenotypes, cells were then cultured for 72 hr in the presence of 20 ng/ml IFN-γ and 100 ng/ml LPS for macrophage M1 polarization, and 20 ng/ml IL-4 and 20 ng/ml IL-13 for macrophage M2 polarization. To determine the differentiation of M2 macrophages by IL-6 signaling, macrophages were pretreated with 100 ng/ml of IL-6 receptor alpha (Abcam, Cambridge, MA, USA) or IL-6 receptor beta (gp130, Cell signaling, Beverly, MA, USA) antibodies and then incubated with TCM for 48 hr.

### Preparation of conditioned medium

To obtain conditioned medium of RWPE-1 cells stimulated with *T*. *vaginalis* as previously reported [31], cells were seeded at 1.5 × 10^5^ cells/well in culture medium in 24-well plates (Corning Inc., Kennebunk, ME, USA). After 24 hr, the medium was changed to serum-free RPMI1640 medium and the cells were incubated with or without live *T*. *vaginalis* in a ratio of 1:10 (1.5 × 10^6^) for 3 hr. After incubation, the supernatant was harvested and clarified with syringe filters (0.2 μm, GVS, Sanford, ME, USA). The supernatants of RWPE-1 cells incubated with or without trichomonads were named trichomonad-conditioned medium (TCM) and conditioned medium (CM), respectively.

In order to prepare conditioned medium of THP-1-derived macrophages treated with CM or TCM, THP-1 cells were seeded at 1.5 × 10^6^ cells/well in culture medium containing 100 nM PMA on 24 well plates. After 24 hr, the PMA-containing medium was removed, and replaced with CM or TCM for another 72 hr. After incubation, supernatants were harvested and clarified with syringe filters (0.2 μm, GVS). Supernatants of THP-1-derived macrophages incubated in medium including 20% CM and TCM were named macrophage-trichomonad conditioned medium (M-TCM) and macrophage-conditioned medium (M-CM), respectively.

### ELISA assays

To measure various inflammatory cytokines (CCL2, CXCL8, IL-1β, TNF-α, IFN-γ, IL-6, IL-4, IL-10, and TGF-β) contained in conditioned medium of RWPE-1 cells stimulated with *T*. *vaginalis*, RWPE-1 cells were seeded at 3 × 10^4^ cells/well on 96-well plates with complete K-SFM medium and incubated for stabilization for 24 hr. The medium was then replaced with fresh serum-free RPMI1640 medium and the cell were incubated with live *T*. *vaginalis* (R:T = 1:10) for 3 hr. After incubation, the supernatant was collected and stored at -20°C.

To assess phenotypic changes in human macrophages induced by conditioned medium of RWPE-1 cells stimulated with *T*. *vaginalis* (TCM), human monocytes were differentiated to M0, M1 or M2 macrophages by the above methods. PMA or M-CSF-induced macrophages were cultured with 5 ng/ml recombinant human (rh) IL-6 protein or 20% conditioned medium (CM or TCM) for 72 hr. To investigate the role of IL-6 on the polarization of the macrophages, cells were pretreated with anti-gp130 antibody (100 ng/ml, Cell signaling) or Ruxolitinib as JAK inhibitor (1 μM, Merck Inc., Eugene, OR, USA) for 1 hr before incubation with TCM. Supernatants were harvested and stored at -20°C. CCL2, CXCL8, and IL-1β, TNF-α, IFN-γ, IL-6, IL-4, IL-10, IL-12, CXCL10 and TGF-β (BD Biosciences Inc., San Jose, CA, USA) were measured by ELISA kits according to the manufacturer’s instructions. The data are expressed as means ± SD of four independent experiments.

### Chemotaxis assays

To measure THP-1 monocyte migration, chemotaxis assays were performed in 24-well microplates. The lower wells were filled with 350 μl of culture medium supplemented with 100 ng/ml recombinant human (rh) CCL2, rhCXCL8 or rhIL-6 (Prospec, East Brunswick, NJ, USA), CM or TCM. Polyvinylpyrrolidone-free polycarbonate filters (Merck Millipore, Tullagreen, Ireland) with 8 μm pores were placed over the lower wells. To promote adhesion of the cells, the filters were pretreated with 100 μg/mL human plasma fibronectin (R&D Systems, Minneapolis, MN, USA) overnight at 4°C and air-dried for 30 min. The upper wells were filled with 200 μl of THP-1 monocytes (2 × 10^5^) in RPMI containing 10% FBS. The plates were incubated for 4 hr at 37°C, the filters were removed, and the cells adhering to their upper surfaces were removed with filter wipes. The lower surfaces were dried, fixed, and stained with Giemsa, and the cells in four randomly selected fields per well were counted under a light microscope. The chemotactic index was calculated from the number of cells that migrated in response to the control. To investigate the contributions of IL-6 to the chemotactic responses of the monocytes, conditioned medium was pretreated with rabbit anti-human IL-6 antibodies (100 ng/mL, Abcam). The data are expressed as means ± SD of four independent experiments.

### Proliferation Assay

To examine the effect of conditioned media on their growth, macrophages were seeded at 2 × 10^4^ cells/well in 96-well plates and incubated in complete culture medium containing 100 nM PMA. After 24 hr, the plates were washed and cultured in serum-free medium, CM or TCM (20%) up to 72 hr. To investigate the effects of IL-6 on proliferation, macrophages were pretreated with anti-gp130 antibody (100 ng/ml, Cell Signaling, Beverly, MA, USA) or Ruxolitinib as a JAK inhibitor (1 μM, Merck Inc., Eugene, OR, USA) for 1 hr, and the plates were incubated with 20% CM or TCM up to 72 hr. Cell growth was analyzed by CCK-8 assays: CCK-8 reagent (Enzo Life Sciences, Farmingdale, NY, USA) was added to each well and the plates were incubated for 2 hr. Absorbance was measured at 450 nm using a microplate reader (Bio-Rad, Richmond, CA, USA).

To determine the effect of M-TCM on prostate cancer cell proliferation, PC3, DU145 and LNCaP were seeded at 1 × 10^4^ cells/well in 96-well plates and incubated in RPMI1640 with 10% FBS. After 24 hr, cells 25% CM, TCM, M-CM or M-TCM was added for 24 hr, and growth was analyzed by CCK-8 assays. Data are expressed as means ± SD of four independent experiments.

### Wound healing assay

We assessed the migration of prostate cancer cells with wound healing assays. PC3, DU145 and LNCaP were seeded at 2 × 10^5^ cells/well in 24-well plates and cultured in RPMI1640 with 10% FBS. When the cells reached confluence they were wounded by scraping across the surface of the well with a sterile micropipette tip. The cells were washed immediately and the wells were filled with serum-free medium or 25% conditioned media (CM, TCM, M-CM, M-TCM, and αgp130+T-TCM) and incubated or 24 hr. Before and after incubation at least five different fields of the wounded area of each sample were photographed using an inverted microscope (Leica Microsystems Inc., Buffalo Grove, IL, USA). Wound areas were measured with ImageJ software (NCI, Bethesda, MD, USA). The percent of each wounded area filled by cell migration was calculated as: {(mean wounded breadth–mean remaining breadth)} / mean wounded breadth × 100. The data are expressed as means ± SD of four independent experiments.

### Real-time quantitative PCR

Total RNA was extracted using Tri-RNA Reagent (Favorgen, Kaohsiung, Taiwan). Concentrations of RNA were determined and quantified by measuring absorbance at 260 and 280 nm with a spectrophotometer. Complementary DNA (cDNA) was synthesized from total RNA using a Maxime RT PreMix kit (iNtRON, Bio Inc, Sungnam, Korea). Real-time PCR analysis was performed with SYBR Green Master Mix on a LightCycler 480 System (Roche, Mannheim, Germany). Primers used are listed in S1 Table. PCR conditions were forty-five cycles at 95°C for 5 min, followed by 95°C for 10 sec, 60°C for 10 sec and 72°C for 10 sec. mRNA expression were quantified in triplicate. Data were measured with LightCycler 480 Software (Roche). GAPDH and β-actin were used as internal controls. The data are expressed as means ± SD of four independent experiments.

### Reverse transcriptase PCR

Total RNA was reverse transcribed to cDNA with a Maxime RT PreMix kit (iNtRON, Bio Inc, Sungnam, Korea). The primers used for cyclin D1, c-Myc and Bcl-2 are presented in S1 Table. PCR conditions were: initial DNA denaturation at 94°C for 2 min, and 30 rounds of denaturation (94°C for 20 sec), annealing (55°C for 10 sec) and extension (72°C for 30 sec). PCR products were electrophoresed on 2% agarose gels containing 0.5 μl/ml ethidium bromide, and photographed under ultraviolet light. β-actin and GAPDH were used as internal controls. The data are expressed as means ± SD of three independent experiments.

### Western blot analysis

Cells were harvested and lysed in PRO-PREP protein extraction solution (iNtRON, Bio Inc, Sungnam, Korea). Protein concentrations were measured with a Bradford Protein Assay Reagent kit (Bio-Rad, Richmond, CA, USA). Proteins were fractionated by 10% SDS-polyacrylamide gels electrophoresis (PAGE), and transferred onto polyvinylidene difluoride (PVDF) membranes. These were incubated with anti-PCNA, anti-cyclin D1, anti-Bcl-2, anti-phospho-JAK2, anti-β-actin Ab (1:1000; Abcam), anti-gp130, anti-phospho-STAT3 and anti-iNOS antibodies (1:1000; Cell Signaling Technology, Beverly, MA, USA) as primary antibodies. Goat anti-rabbit horseradish peroxidase-conjugated IgG or goat anti-mouse horseradish peroxidase-conjugated IgG (Abcam, Cambridge, MA, USA) served as secondary antibodies. Protein bands were detected with a chemiluminescence reagent kit (SurModics, MN, USA). The data are expressed as means ± SD of three independent experiments.

### Immunofluorescence assay

Cells were washed, fixed with 4% paraformaldehyde for 10 minutes at −20°C and blocked with 0.1% normal goat serum for 1 hour. The cover glasses were then incubated with anti-c-Myc antibody or anti-IL-6 receptor antibody (1:50, rabbit polyclonal, Bioss Inc, Woburn, MA, USA) overnight at 4°C, and then washed and stained with Alexa 594-labelled goat anti-rabbit lgG (#A11012, 1:500, Invitrogen, CA, USA) at 37°C for 1 hr. The cover glasses were mounted in Vectashield mounting medium (Vector Laboratories, Burlingame, CA) with DAPI to visualize nuclei. Images photographed by fluorescence microscope (Leica, Las software).

### In vivo study

To test whether *T*. *vaginalis* infection induces accumulation of M2 macrophage in prostate cancer tissue *in vivo*, a total of 10 male C57BL/6 mice (6-weeks-old) were purchased from Koatech (Pyeongtaek, Gyeonggido, Korea) and maintained in laminar flow cabinets under specific pathogen-free conditions. The animals had free access to sterilized food and water and were housed in a regulated environment with a 12 hr reversed light and dark cycle in an approved experimental laboratory. For inoculation of tumor cells as previously reported [32, 33], mice were anaesthetized by isoflurane inhalation and placed in the supine position. TRAMP-C2 cells (mouse prostate cancer cell line, 2 × 10^6^ cells) without or with live *T*. *vaginalis* (2 × 10^5^ cells) were suspended in 0.1 ml PBS mixed with Matrigel. A lower midline abdominal incision was made and the tumor cell suspension was injected into the prostate using a 30-gauge needle and a 1-ml disposable syringe. The surgical wound was closed with two layers of 4–0 Dexon interrupted sutures. After four weeks, all mice were killed by CO_2_ exposure. A midline incision was made to access to the abdominal cavity and the prostates were harvested.

### Immunohistochemistry assay

Prostate tissues were harvested, fixed in 10% buffered neutral formalin, embedded in paraffin blocks and processed by standard protocols for immunohistochemistry assays (IHC). Sections were incubated with anti-CD206 antibodies (1:1000; Abcam, Cambridge, MA, USA) for 1 hr at room temperature. The sections were treated with biotin-conjugated anti-rabbit antibody and visualized with 3.3’-diaminobenzidine (DAB). Slides were counterstained with hematoxylin, and images were taken with a Leica DM2000 microscope equipped with a DFC425 digital camera and Leica Application Suite v3.8.0. The immunostaining was reviewed by the two pathologists blinded to the study design. In evaluating IHC results, each value was recorded as a numerical score from 0 to 10 based on proportion of clusters of immunopositive cells.

### Statistical analysis

All data are expressed as means ± standard deviations (SD) of three or four independent experiments. Comparisons were made using the one or two-tailed Mann-Whitney U-test with Prism 6.0 software (GraphPad Inc.), and *P*-values < 0.05 were considered statistically significant.

## Results

### Production of cytokines by human prostate epithelial cells (RWPE-1) stimulated with *T*. *vaginalis*

**Fig 1:**
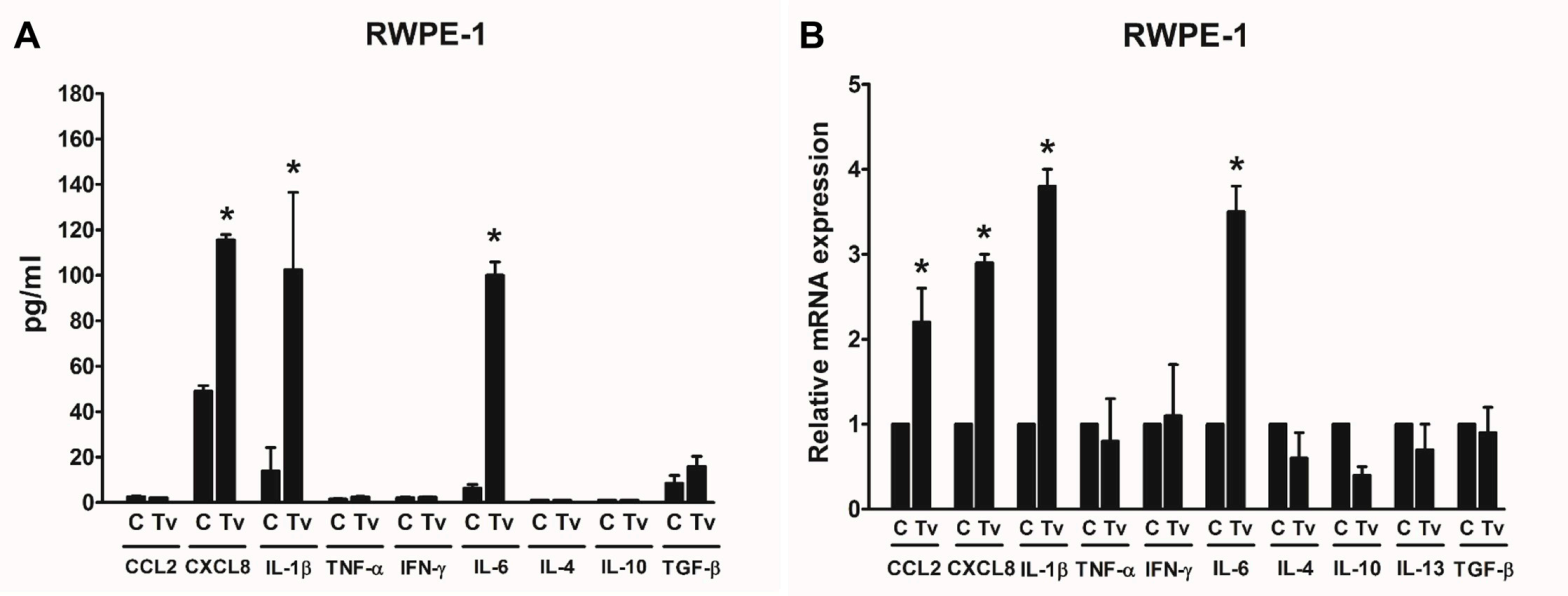
Production of cytokines by human prostate epithelial cells (RWPE-1) stimulated with *T*. *vaginalis*. **(A)** RWPE-1 cells were incubated with *T*. *vaginalis* (R:T = 1:10) for 3 hr. Production of cytokines was determined by ELISA assays. **(B)** Cytokine mRNAs were determined by real-time PCR. Data are means ± SD of four independent experiments. *p<0.05 versus untreated RWPE-1 (C).

Inflammation is a key component of the tumor microenvironment and has been implicated in the pathogenesis of various tumor types. Tumor-associated macrophages (TAMs) are key components of the link between inflammation and cancer [34, 35]. In previous studies, *T*. *vaginalis* infection induced the production of inflammatory cytokines such as IL-1β, IL-6, CCL2 and CXCL8 in prostate epithelial cells as well as cervical cancer cells [31, 36, 37]. In this study, we measured whether *T*. *vaginalis*-stimulated prostate epithelial cells produce Th1 cytokines such as TNF-α and IFN-γ or Th2 cytokines such as IL-6, IL-4, IL-10, IL-13 and TGF-β, which may influence on the differentiation of M1 or M2 macrophage, respectively. As a result, prostate epithelial cells stimulated with *T*. *vaginalis* increased only IL-6 production among these Th2 cytokines (Fig 1). In addition, CCL2, CXCL8 and IL-1β are known to induce the migration of monocytes. IL-1β and CXCL8 were increased in both protein and mRNA levels by stimulation of RWPE-1 cells with *T*. *vaginalis* (Fig 1). On the other hand, CCL2 was increased only in mRNA but not in protein levels (Fig 1B). Therefore, our results suggest that cytokines of prostate epithelial cells produced by *T*. *vaginalis* infection may affect macrophage differentiation as well as monocyte migration.

### Migration of monocytes in response to TCM

**Fig 2:**
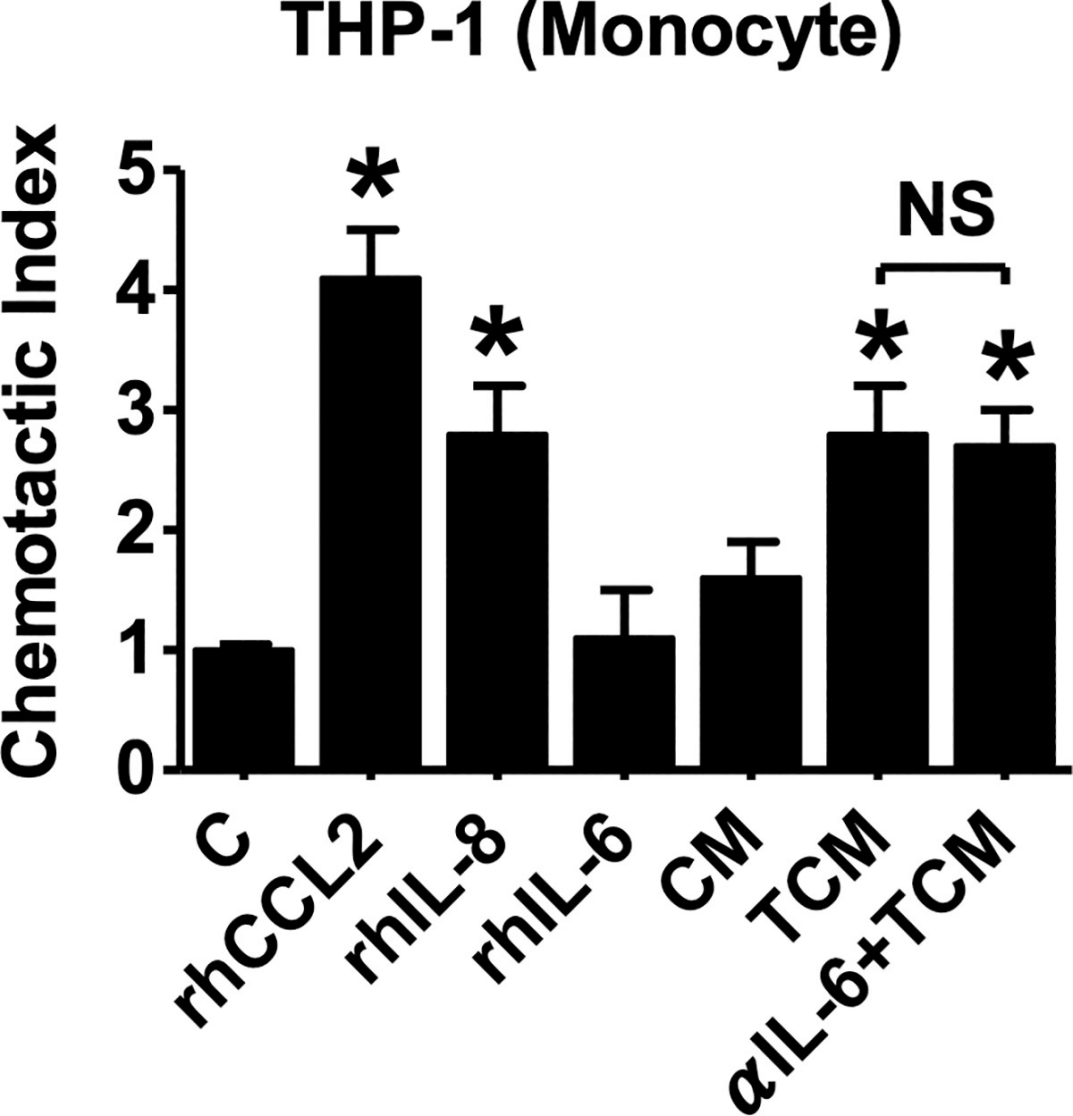
Migration of monocytes (THP-1) induced by conditioned medium of human prostate epithelial cells (RWPE-1) stimulated with *T*. *vaginalis*. THP-1 cells (1 × 10^5^) were added to the upper compartment of Boyden chambers, and the following chemicals or conditioned media were added to lower chambers; recombinant human (rh) CCL2 (100 ng/ml), rhCXCL8 (100 ng/ml), rhIL-6 (100 ng/ml), conditioned medium of RWPE-1 cells alone (CM), conditioned medium of RWPE-1 cells stimulated with *T*. *vaginalis* (TCM), or TCM preincubated with 100 ng/ml of neutralizing antibody to IL-6. After 6 hr, cells that had migrated to the lower chambers were counted. Data are means ± SD of four independent experiments. *p<0.05 versus untreated THP-1 cells (C). NS = not statistically significant.

TAMs are derived from circulating monocytes, which are selectively attracted to the tumor microenvironment by locally produced chemotactic factors [34, 38]. We investigated whether conditioned medium of RWPE-1 cells stimulated with *T*. *vaginalis* (TCM) attracted monocytes. TCM increased the migration of THP-1 monocytes 2.8 fold compared to control. When rhCCL2 and rhCXCL8 proteins were used as positive controls, the monocyte migration increased by 4.1 and 2.8 fold, respectively, compared to control. To assess the involvement of IL-6 in the chemotactic effect on THP-1 monocytes, we placed rhIL-6, or TCM pretreated with anti-IL-6 antibody, in the lower wells of Boyden chambers. Migration of the monocytes was not affected by IL-6, and blockade of IL-6 by antibody had no effect on TCM-induced monocyte migration (Fig 2). These results suggest that chemokines such as CCL2 and CXCL8 contained in TCM, but not IL-6, stimulate monocyte recruitment.

### Polarization of PMA-THP-1 macrophages into the M2-like macrophage phenotype is induced by TCM

**Fig 3:**
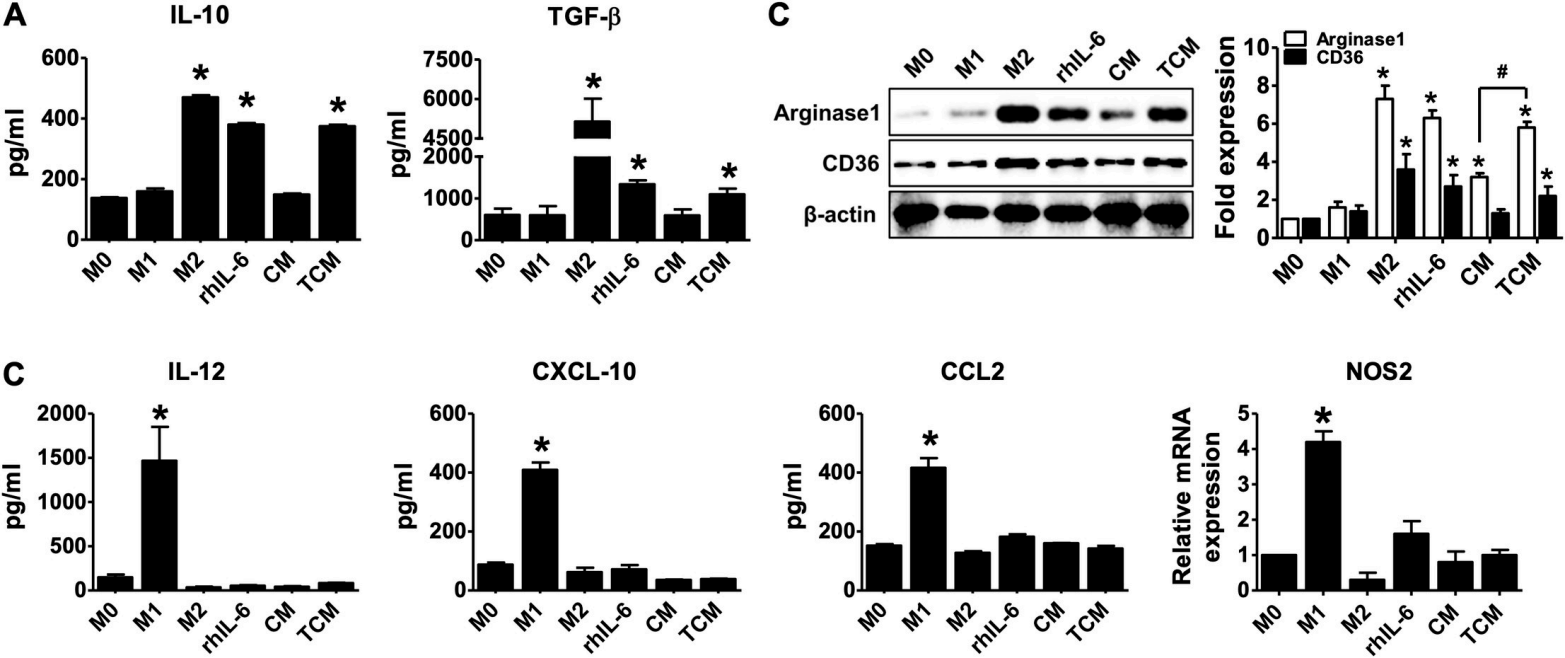
Polarization of PMA-THP-1 macrophages to the M2-like macrophage phenotype by conditioned medium of human prostate epithelial cells (RWPE-1) stimulated with *T*. *vaginalis*. THP-1 monocytes were treated with 100 nM PMA for 24 hr and then cultured in absence of PMA for 72 hr (M0). The cells were then treated with 100 ng/ml LPS and 20 ng/ml rhIFN-γ for M1 macrophages (M1), 20 ng/ml rhIL-4 and 20 ng/ml rhIL-13 for M2 macrophages (M2), 5 ng/ml rhIL-6, CM, or TCM for 72 hr. IL-10, TGF-β, CD36 and arginase-1 was determined as M2 macrophage markers. **(A)** Production of IL-10 and TGF-β measured by ELISA assays. **(B)** Expression of CD36 and arginase-1 was determined by western blot. Graph represent densitometric analysis (means of three independent western blot experiments). **(C)** IL-12, CXCL-10, CCL2 and NOS2 were measured as M1 macrophage markers. Production of IL-12, CXCL-10 and CCL2 measured by ELISA assays. Expression of *NOS2* was analyzed by real-time PCR. Data are means ± SD of three independent experiments. *p<0.05 versus THP-1-derived macrophage (M0). CM: conditioned medium of RWPE-1 alone, TCM: conditioned medium of RWPE-1 stimulated with *T*. *vaginalis*.

IL-6 induces macrophages to polarize alternatively activated macrophages [39, 40]. In order to determine whether TCM induces polarization of THP-1-derived macrophages to M2, macrophages (M0) induced by PMA were treated with TCM, and identified by measuring the expression of M2 macrophage markers such as IL-10, TFG-β, CD36 and arginase-1 and the expression of M1 macrophage markers such as IL-12, CXCL10, CCL2 and NOS2. TCM increased the M2 markers such as IL-10, TGF-b, CD36 and arginase 1 with THP-1-derived macrophage treated with rhIL-4 and rhIL-13 for M2 macrophage differentiation (Fig 3A), on the other hand, had no effect on the M1 markers (Fig 3B). In addition, THP-1-derived macrophage treated with rhIL-6 also resulted in increased markers of M2 macrophage (Fig 3).

### Polarization of *human* monocyte-derived macrophages into M2-like macrophage phenotypes is induced by TCM

**Fig 4:**
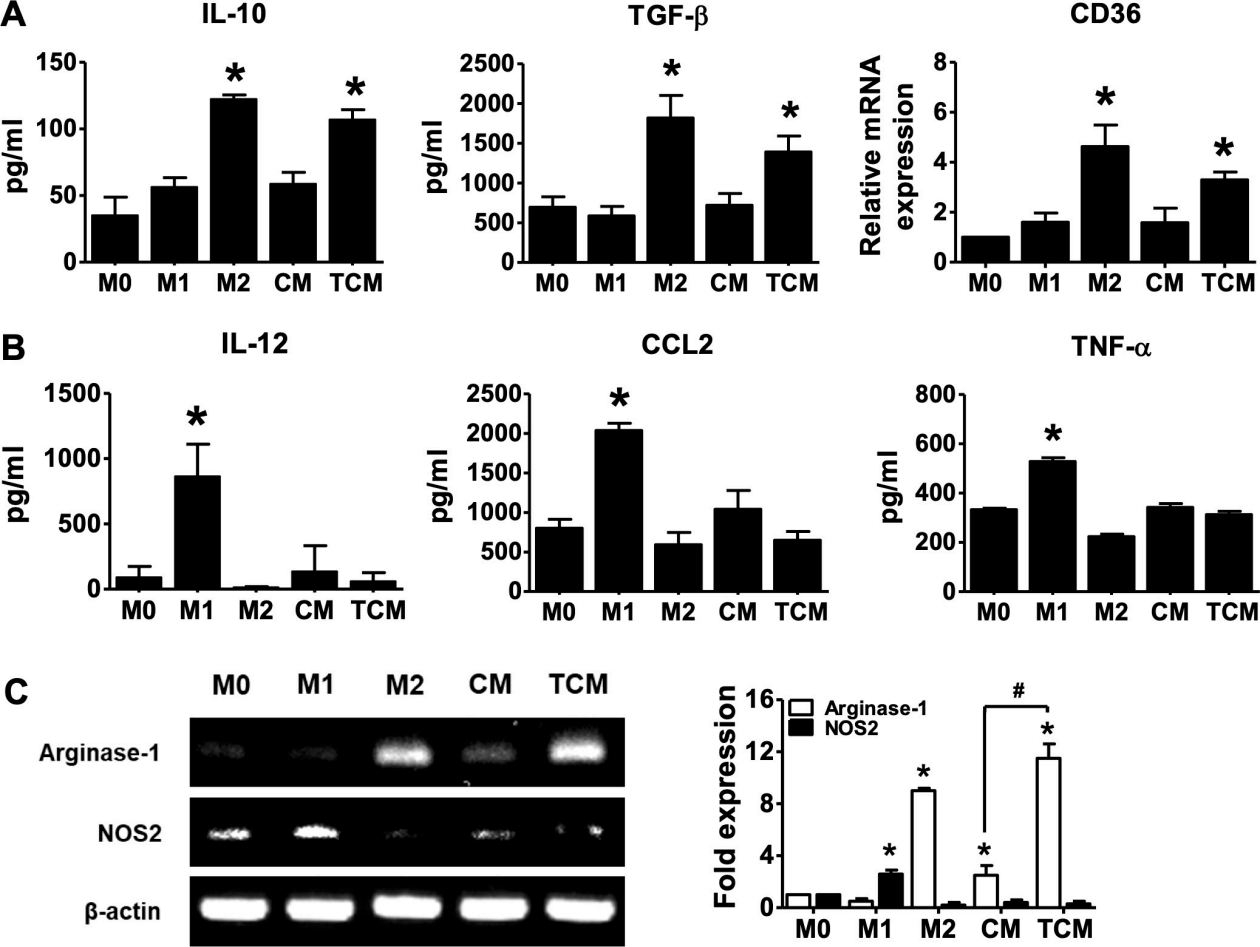
Polarization of human monocyte-derived macrophages (HMDM) to the M2-like macrophage phenotype is induced by conditioned medium of human prostate epithelial cells (RWPE-1) stimulated with *T*. *vaginalis*. Human peripheral blood mononuclear cells (PBMCs) incubated with 50 ng/ml M-CSF for 2 hr to allow monocyte adhesion. Non-adherent cells were removed and adherent monocytes were treated with 50 ng/ml M-CSF for 6 days for macrophage differentiation, and then cultured with 100 ng/ml LPS and 20 ng/ml rhIFN-γ for M1 macrophages (M1), 20 ng/ml rhIL-4 and 20 ng/ml rhIL-13 for M2 macrophages (M2), CM, or TCM for 48 hr. Macrophages were identified with M1 markers such as IL-12, CCL2, TNF-α and NOS2, and M2 markers such as IL-10, TGF-β, CD36 and arginase-1. **(A)** Production of IL-10 and TGF-β was measured by ELISA assay. Expression of *CD36* was evaluated by real-time PCR. **(B)** Production of IL-12, CCL2 and TNF-α was measured by ELISA assay. **(C)** mRNA expression of *arginase-1* and *NOS2* were detected by RT-PCR. Densitometry of mRNA bands were quantified by three independent experiments. Data are means ± SD of three independent experiments. *p<0.05 versus HMDM (M0). CM: conditioned medium of RWPE-1 cells alone, TCM: conditioned medium of RWPE-1 cells stimulated with *T*. *vaginalis*.

To test for polarization of human macrophages into the M2-type by TCM, human PBMCs-derived macrophages were treated with TCM. Markers of M2 macrophages such as IL-10, TGF-β, CD36 and arginase-1 were significantly increased by TCM, as well as by rhIL-4 plus rhIL-13 (Fig 4A and 4C), while markers of M1 macrophages such as IL-12, CCL2, TNF-*α* and NOS2 were not increase (Fig 4B and 4C). These results show that supernatants of RWPE-1 cells co-cultured with *T*. *vaginalis* induce the differentiation of macrophages to an M2-like phenotype.

### Proliferation of human macrophages in response to TCM

**Fig 5:**
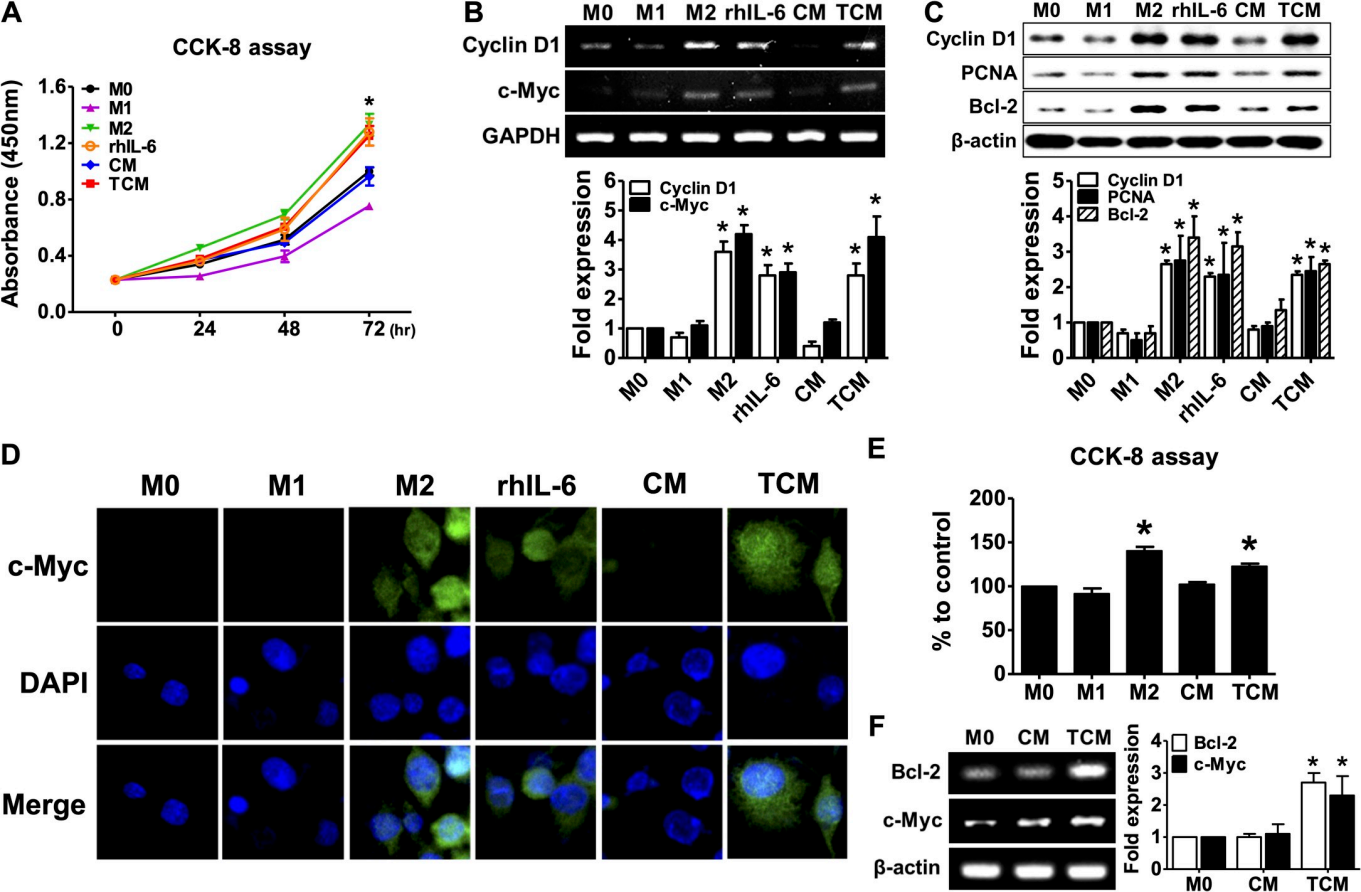
Proliferation of macrophages in response to conditioned medium of human prostate epithelial cells (RWPE-1) stimulated with *T*. *vaginalis*. THP-1 monocytes were treated with 100 nM PMA for 24 hr to obtain M0 macrophages and then cultured in complete medium for 72 hr. The cells were then treated with 100 ng/ml LPS and 20 ng/ml IFN-γ for M1 macrophages, 20 ng/ml IL-4 and 20 ng/ml IL-13 for M2 macrophages, 5 ng/ml IL-6, CM, or TCM for 72 hr. Human peripheral blood mononuclear cells (PBMCs) incubated with 50 ng/ml M-CSF for 2 hr to allow monocyte adhesion. Non-adherent cells were removed and adherent monocytes were treated with 50 ng/ml M-CSF for 6 days for macrophage differentiation, and then cultured with CM, or TCM for 48 hr. **(A)** Proliferation of THP-1-derived macrophages was measured by CCK-8 assay. **(B)** mRNA expression of *cyclin D1* and *c-Myc* was determined by RT-PCR. Densitometry of mRNA bands were quantified by three independent experiments. **(C)** Expression of PCNA, cyclin D1 and Bcl-2 was determined by western blot. Graph represent densitometric analysis (means of three independent western blot experiments). **(D)** Immunofluorescence assays were performed using an anti-c-Myc primary antibody and an anti-rabbit Alexa Fluor-594-labeled secondary antibody (DNA stained with DAPI/blue) and images were selected from five random fields. **(E)** Proliferation of human PBMCs-derived macrophages was measured by CCK-8 assay. **(F)** Expression of *Bcl-2* and *c-Myc* was determined by RT-PCR. Densitometry of mRNA bands were quantified by three independent experiments. Data are means ± SD of three independent experiments. *p<0.05 versus THP-1-derived macrophages or HMDM (M0). CM: conditioned medium of RWPE-1 alone, TCM: conditioned medium of RWPE-1 stimulated with *T*. *vaginalis*.

M2 macrophages were known to proliferate in response to Th2 inflammation [27, 28, 41]. To determine whether TCM induces proliferation of human macrophages, THP-1-derived macrophages and PBMC-derived macrophages were incubated with TCM. As shown in Fig 5A, proliferation of THP-1-derived macrophages was significantly increased by rhIL-4 plus rhIL-13 (M2), rhIL-6 and TCM, while LPS plus rhIFN-γ (M1) resulted in decreased proliferation of macrophages than M0. Expression of proliferative signals such as cyclin D1, c-Myc, PCNA and Bcl-2 was increased by rhIL-4 plus rhIL-13 (M2), rhIL-6 and TCM (Fig 5B–5D).

In addition, proliferation of PBMC-derived macrophages was significantly increased by TCM (Fig 5E), and expression of *Bcl-2* and *c-Myc* mRNA was also increased (Fig 5F). Thus, we suggest that TCM induces the proliferation of macrophages as a feature of M2 macrophages.

### Activation of the IL-6R/JAK2/STAT3 signaling pathway is induced by TCM in THP-1-derived macrophages

**Fig 6:**
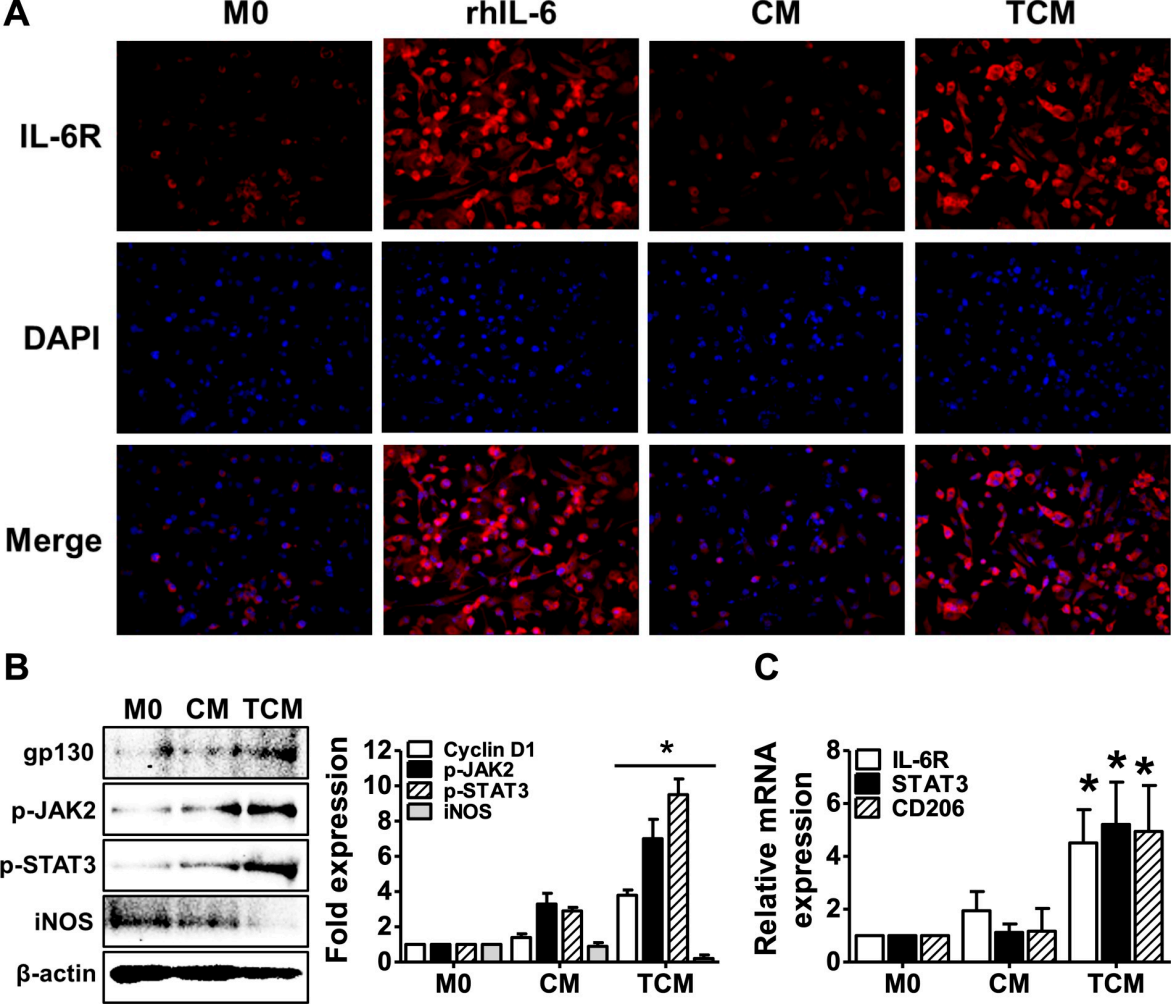
Activation of the IL-6R/JAK2/STAT3 signal pathway in THP-1-derived macrophages is induced by conditioned medium of human prostate epithelial cells (RWPE-1) stimulated with *T*. *vaginalis*. THP-1 cells were treated with 100 nM PMA for 24 hr, and cultured with rhIL-6 (5 ng/ml), CM, or TCM for 24 hr. **(A)** Expression of IL-6 receptor was examined by immunofluorescence using an anti-IL-6R primary antibody and an anti-rabbit Alexa Fluor-594-labled secondary antibody and detected three randomly chosen fields. **(B)** Gp130, p-JAK2, p-STAT3 and iNOS were determined by western blot. Graph represent densitometric analysis (means of three independent western blot experiments). **(C)** Expression of *IL-6R*, *STAT3* and *CD206* mRNA was evaluated by real-time PCR. Data are means ± SD of three independent experiments. *p<0.05 versus THP-1-derived macrophages (M0). CM: conditioned medium of RWPE-1 alone, TCM: conditioned medium of RWPE-1 stimulated with *T*. *vaginalis*.

IL-6 signaling have been reported to promote polarization to alternative activated macrophages, and to induce an anti-inflammatory response in macrophages [26, 42]. To determine whether TCM (containing IL-6) induces activation of the IL-6R/JAK2/STAT3 signaling pathway in human macrophages, we studied the expression of the IL-6 receptor, JAK2, STAT3, iNOS and CD206. Expression of the IL-6 receptor as well as gp130, a member of the IL-6 receptor family, was increased by TCM. Expression of p-JAK2 and p-STAT3 was also increased by TCM. However, expression of the M1 macrophage marker, iNOS, was reduced (Fig 6A and 6B). Transcripts of the *IL-6R* and *STAT3* were increased by TCM, and transcripts of *CD206*, the mannose receptor, a known M2 macrophage marker, were significantly increased by TCM (Fig 6C). These results suggest that the IL-6 was contained in the conditioned medium of RWPE-1 cells and produced in response to *T*. *vaginalis* activates the IL-6R/JAK2/STAT3 signaling pathway along with M2 polarization of the THP-1-derived macrophages.

### Involvement of IL-6 signaling in M2-type macrophage polarization of THP-1 cells induced by TCM

**Fig 7:**
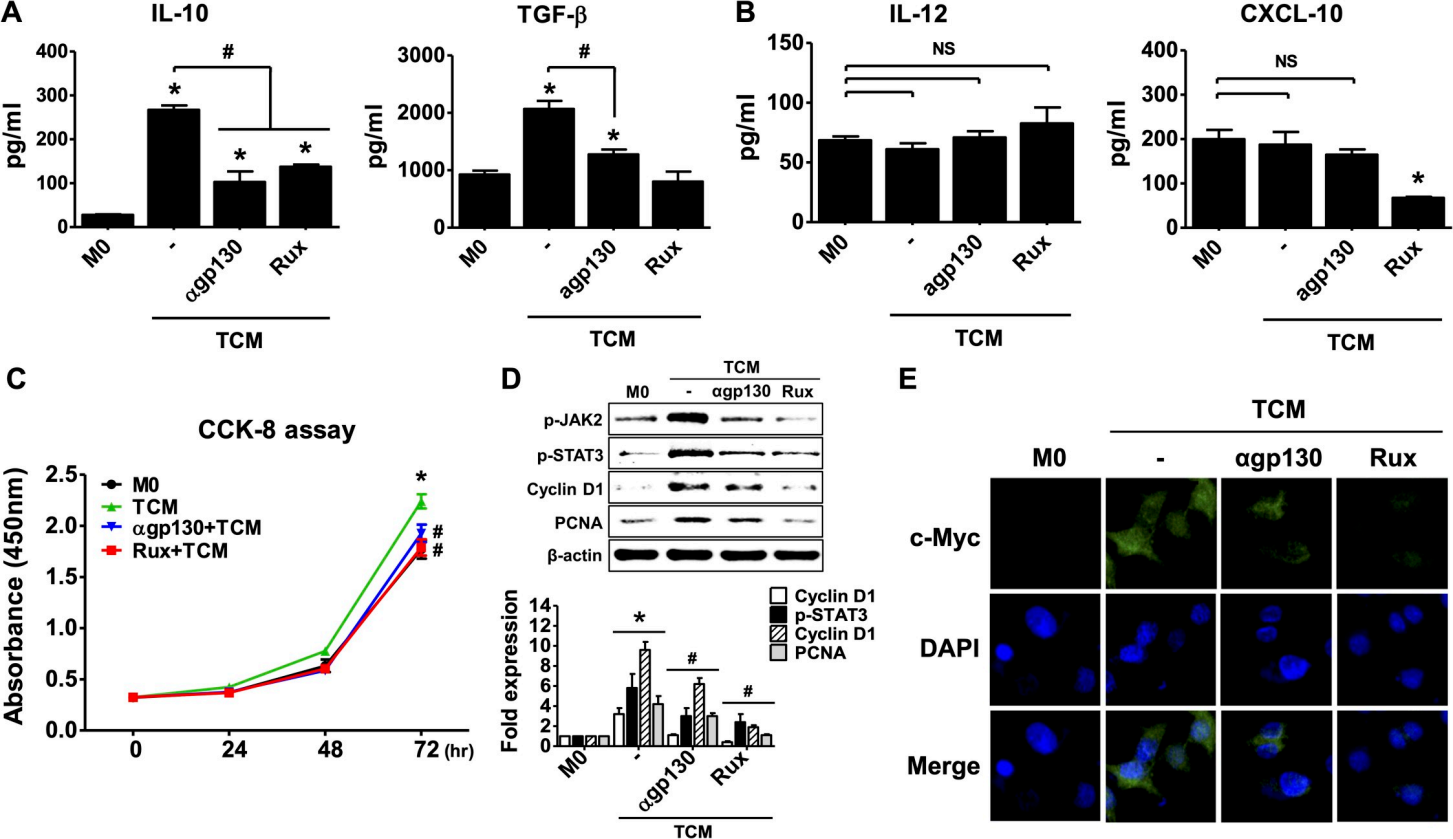
Involvement of the IL-6 signaling pathway in M2-type macrophage polarization of THP-1 cells. THP-1 cells were treated with 100 nM PMA for 24 hr, and cultured with CM, or TCM for 72 hr. To examine involvement of IL-6 signaling in M2 macrophage polarization, M0 macrophages were pretreated with anti-gp-130 antibody (100 ng/ml) or ruxolitinib (JAK inhibitor, 10 μM) before addition of TCM. **(A)** Production of IL-10 and TGF-β as M2 macrophage markers was measured by ELISA assay. **(B)** Production of IL-12 and CXCL-10 as M1 macrophage markers was measured by ELISA assays. **(C)** Proliferation of macrophages was evaluated by CCK-8 assays. **(D)** Expression of p-JAK2, p-STAT3, cyclin D1 and PCNA protein was determined by western blot. Graph represent densitometric analysis (means of three independent western blot experiments). **(E)** Immunofluorescence assays were performed using an anti-c-Myc primary antibody and an anti-rabbit Alexa Fluor-594-labled secondary antibody and images selected from five random fields. Data are means ± SD of three independent experiments. *p<0.05 versus THP-1-derived macrophage (M0). #*p*<0.05 versus conditioned medium of RWPE-1 stimulated with *T*. *vaginalis* (TCM). NS = not statistically significant. CM: conditioned medium of RWPE-1 alone, TCM: conditioned medium of RWPE-1 stimulated with *T*. *vaginalis*, Rux: Ruxolitinib.

We examined whether TCM stimulates M2 polarization of macrophages through the IL-6 receptor and JAK signaling pathway. THP-1-derived macrophages were pretreated with anti-gp130 antibody and Ruxolitinib to block the IL-6R-JAK signaling pathway. These pretreatments inhibited the production of IL-10 and TGF-β (Fig 7A), while M1 markers such as IL-12 and CXCL10, did not change (Fig 7B). Macrophage proliferation was also significantly inhibited by anti-gp130 antibody and ruxolitinib (Fig 7C), and up-regulation of p-JAK2, p-STAT3, cyclin D1 and PCNA expression was inhibited (Fig 7D). Expression of c-Myc decreased (Fig 7E).

In addition, antibody against IL-6 receptor alpha, which is no cross-reactivity to other cytokines, also inhibited the differentiation of M2 macrophages by TCM. It was similar to the results of treatment with gp130 antibody (S1 Fig). These results show that conditioned medium of RWPE-1 cells produced by *T*. *vaginalis* induces M2 polarization of THP-1-derived macrophage via IL-6 acting through the IL-6R/JAK2/STAT3 signaling pathway.

### Accumulation of M2 macrophages in prostate tumor tissue of mice injected with *T*. *vaginalis*

**Fig 8:**
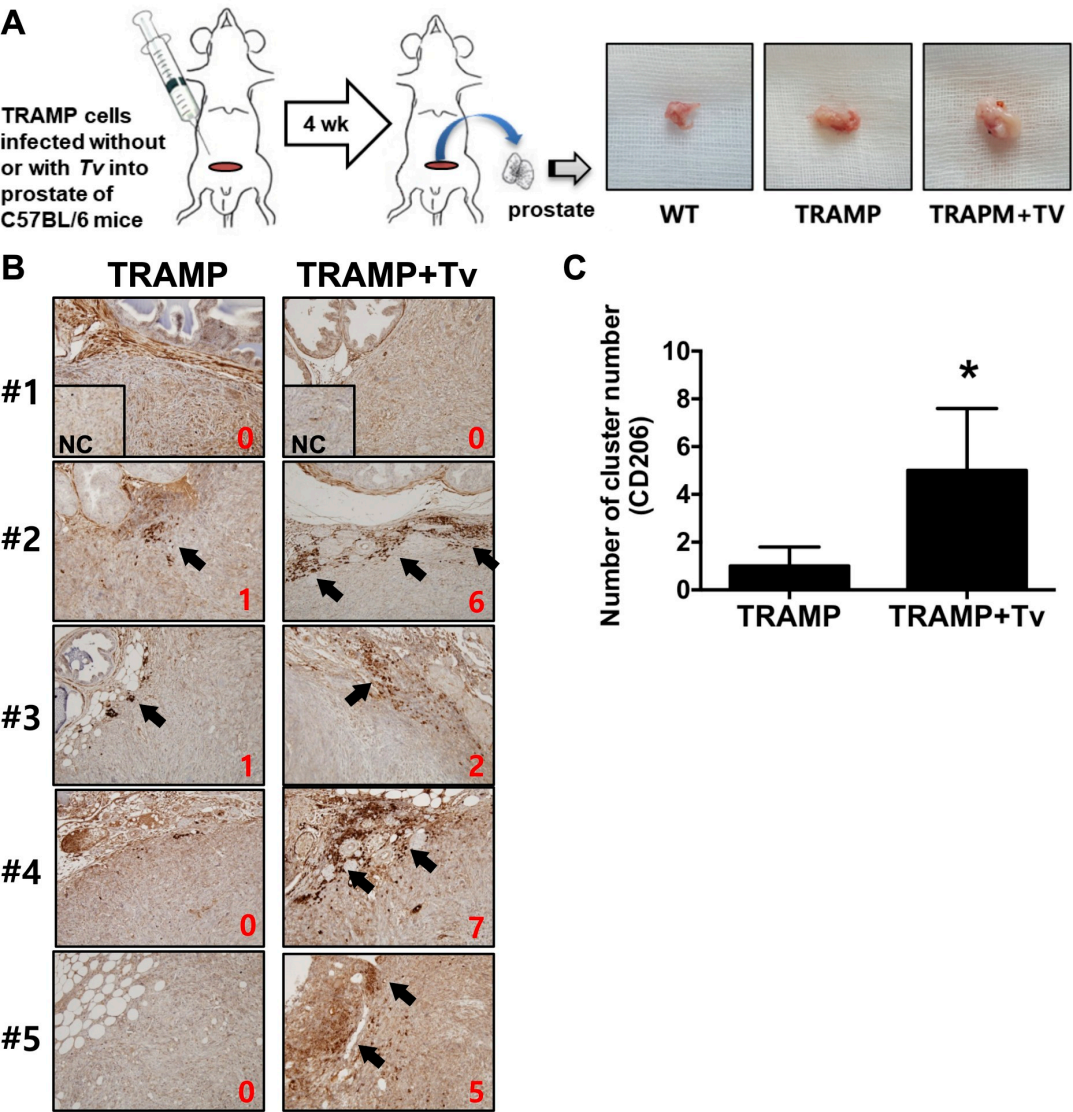
Increased CD206 expression in prostate tissue is induced by TRAMP-C2 cells co-injected with *T*. *vaginalis*. C57BL/6 mice (n = 5/group) were injected in the prostate with TRAMP-C2 mouse cancer cell line cells (without or with live *T*. *vaginalis*). After four weeks, prostate tissues were harvested. (A) Experimental scheme for the animal study. Representative macroscopic images show the resected prostate tissues from mice. (B) Expression of CD206 was observed by immunohistochemistry (IHC). For negative control (NC), only secondary antibody was reacted (insets). Red number in IHC panels was represented as a numerical score from 0 to 10 based on proportion of clusters of immunopositive cells. (C) Data represented as histograms. Bars, means ± SD of five independent experiments (*p = 0,0317 with the one-tailed Mann-Whitney U test). WT: wild-type, NC: negative control.

To test whether *T*. *vaginalis* infection induces accumulation of M2 macrophages in prostate tumor tissue, TRAMP-C2 cells (from a mouse prostate cancer cell line) were mixed with live *T*. *vaginalis* and injected into the prostates of mice. Expression of CD206, a known marker of M2 macrophages, increased significantly more in the tumor tissue of prostates injected with tumor cells mixed *T*. *vaginalis* than in tissues injected with tumor cells alone (Fig 8). These results suggest that *T*. *vaginalis* infection stimulates the accumulation of M2 macrophages in prostate tumor tissue.

### Proliferation and migration of prostate cancer cells induced by conditioned medium of THP-1-derived macrophage cultured with TCM (M-TCM)

**Fig 9:**
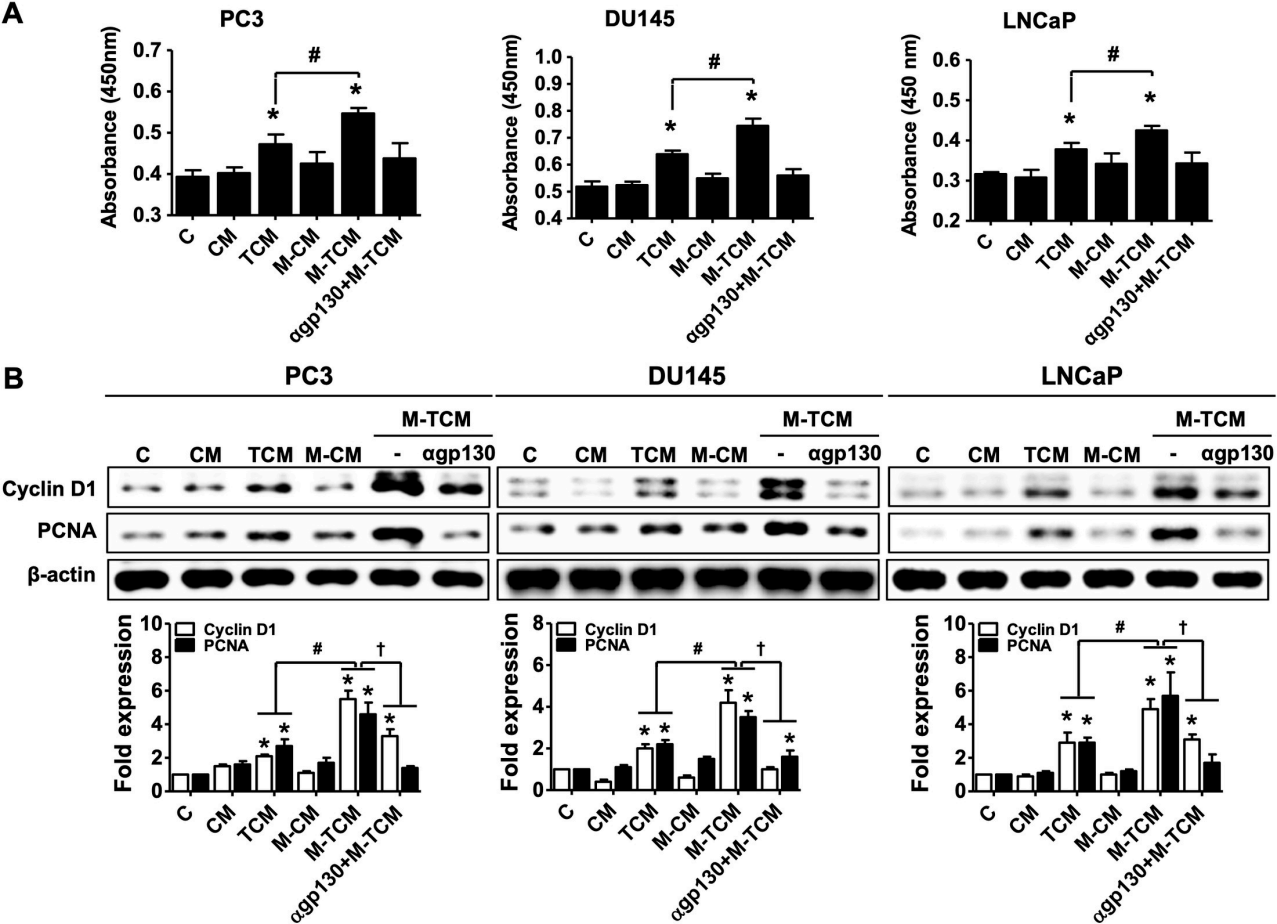
Proliferation of prostate cancer cells in response to conditioned medium of M2-like macrophages cultured with TCM. To prepare the conditioned medium of M2-like macrophages, THP-1-derived macrophages were incubated in RPMI1640 media containing 20% TCM with or without pretreatment of gp130 antibody for 72 hr, and the culture supernatants were collected. The supernatants was named αgp130+M-TCM and M-TCM. Prostate cancer cells (PC3, DU145 and LNCaP) were cultured with CM, TCM, M-CM, M-TCM or αgp130+M-TCM for 24 hr. **(A)** Proliferation of the prostate cancer cells was measured by CCK-8 assay. **(B)** Cyclin D1 and PCNA were determined by western blot. Graph represent densitometric analysis (means of three independent western blot experiments). Data are means ± SD of three independent experiments. *p<0.05 versus untreated prostate cancer cells (C). #*p*<0.05 versus conditioned medium of RWPE-1 stimulated with *T*. *vaginalis* (TCM). †*p*<0.05 versus conditioned medium of RWPE-1 stimulated with *T*. *vaginalis* (TCM). CM: conditioned medium of RWPE-1 alone, TCM: conditioned medium of RWPE-1 stimulated with *T*. *vaginalis*, M-CM: conditioned medium of THP-1-derived macrophage stimulated with CM, M-TCM: conditioned medium of THP-1-derived macrophage stimulated with TCM, αgp130+M-TCM: conditioned medium of THP-1-derived macrophage stimulated with TCM after pretreatment of gp130 antibody.

TAMs are mainly M2 macrophages and play crucial roles in proliferation, survival and metastasis of cancer cells [22, 24]. Here, we asked whether conditioned medium of M2-like macrophages differentiated in response to TCM (M-TCM) induced the progression of prostate cancer including proliferation, survival and cell migration. Progression in response to M-TCM was compared with that of conditioned medium from *Tv-*infected prostate epithelial cells (TCM) to clearly identify the effects of the M2 macrophages on the progression of prostate cancer cells. In this study, we used androgen-independent PC3 and DU145 and androgen-dependent LNCaP as human prostate cancer cell lines. Proliferation of three prostate cancer cells was measured by CCK-8 assays. As shown in Fig 9A, proliferation of the prostate cancer cells was significantly increased by TCM and M-TCM, and M-TCM-treated cells grew more than TCM-treated calls (Fig 9A). Proteins associated with cell survival such as cyclin D1 and PCNA were also induced more by M-TCM than TCM in all three prostate cancer cell lines (Fig 9B).

**Fig 10:**
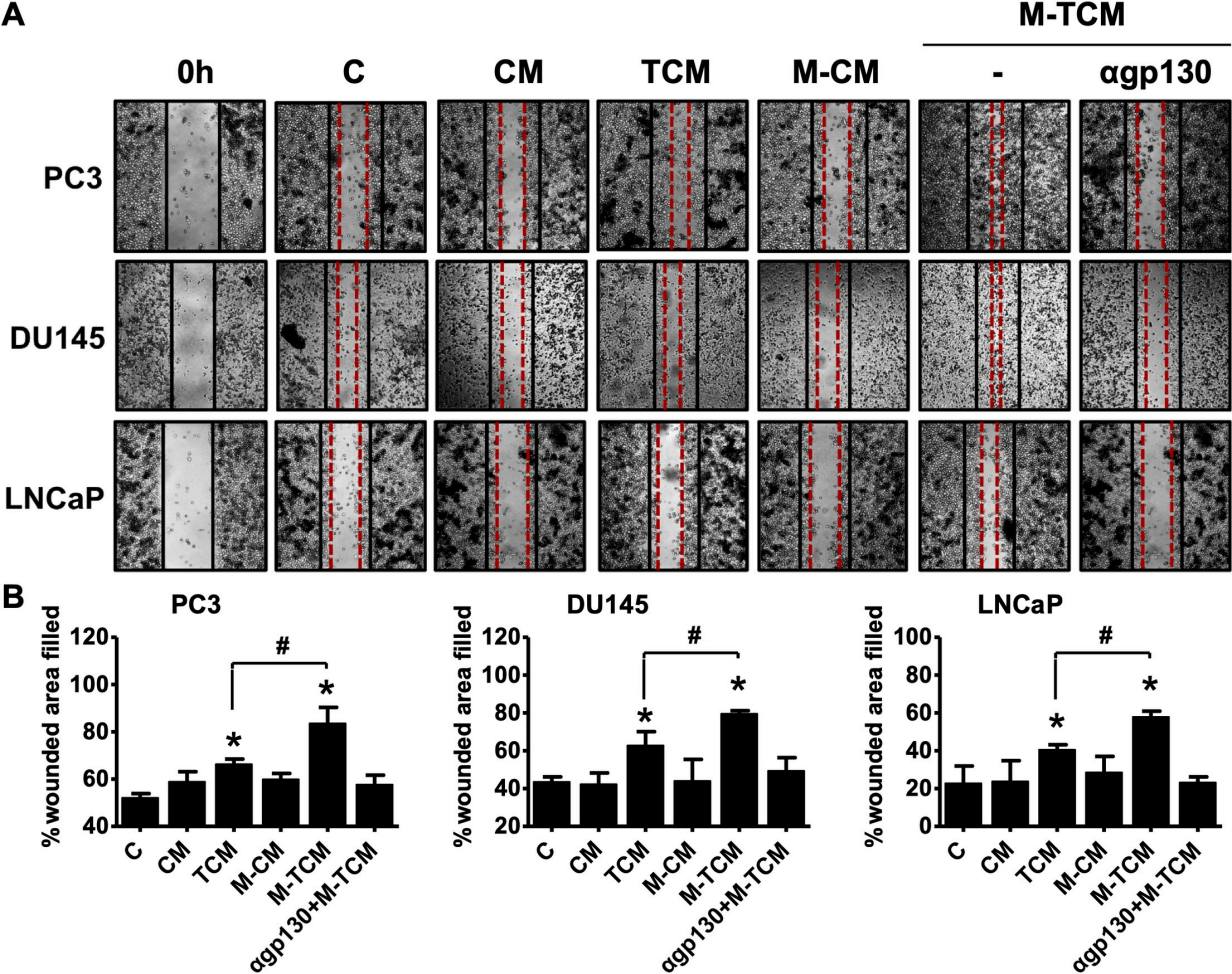
Migration of prostate cancer cells is induced by conditioned medium of M2-like macrophages cultured with TCM. Prostate cancer cells (PC3, DU145 and LNCaP) were cultured with CM, TCM, M-CM, M-TCM or αgp130+M-TCM for 24 hr. **(A)** Migration of the prostate cancer cells was assessed by wound healing assays. Representative images photographed by light microscope. **(B)** Migration was measured as: {(mean wounded breadth–mean remaining breadth)} / mean wounded breadth × 100. Data are means ± SD of three independent experiments. *p<0.05 versus untreated prostate cancer cells (C). #*p*<0.05 versus conditioned medium of RWPE-1 stimulated with *T*. *vaginalis* (TCM). CM: conditioned medium of RWPE-1 alone, TCM: conditioned medium of RWPE-1 stimulated with *T*. *vaginalis*, M-CM: conditioned medium of THP-1-derived macrophages stimulated with CM, M-TCM: conditioned medium of THP-1-derived macrophages stimulated with TCM. αgp130+M-TCM: conditioned medium of THP-1-derived macrophage stimulated with TCM after pretreatment of gp130 antibody.

The migratory ability of the prostate cancer cells was measured by wound healing assays. M-TCM-treated cells of all three prostate cancer cell lines displayed greater migration ability than TCM-treated cells (Fig 10).

In addition, to investigate the effect of M2 macrophage on the proliferation and migration of prostate cancer cells by M-TCM, prostate cancer cells were cultured in a conditioned medium of THP-1-derived macrophages (αgp130+M-TCM) that inhibited the differentiation of M2 macrophages by gp130 antibody. As a result, prostate cancer cells treated with gp130+M-TCM did not increase the proliferation and migration. Thus, our findings indicate that M2-like macrophages induced by TCM enhance the proliferation and migration of prostate cancer cells. However, further research is needed to elucidate the definite role of M2 macrophages in the proliferation and migration of prostate cancer cells by M-TCM.

## Discussion

Prostate cancer is one of the most common cancer and the second leading cause of cancer-related deaths among men in the Western world [43]. It has been reported to be affected by multiple factors including age, race, diet, heredity, and environment [44]. Additionally, inflammation and infection may play an important role in its development [45–47]. At the biological level, chronically inflamed cells play a major role in the tumor microenvironment, which promotes tumor growth and metastasis by producing cytokines such as TNF-α, CCL2, CXCL8, IL-6 and TGF-β and by inducing infiltration of leukocytes comprising innate immune cells including macrophages, neutrophils, mast cells, dendritic cells, and natural killer cells, as well as adaptive immune T cells and B cells [14, 22].

*T*. *vaginalis* is one of most common pathogens causing STD; it is known to induce clinical prostatitis and chronic inflammation, and may contribute to prostate carcinogenesis via symptomatic prostatic inflammation [3, 5]. Recently, we have demonstrated that *T*. *vaginalis* infections through the urethra in rats induce prostatitis [48]. Gardner and colleagues observed inflammatory infiltrates and focal areas of atypical epithelial hyperplasia near *T*. *vaginalis* organisms in prostate tissue from infected men [49, 50]. Also, Sutcliffe et al reported a positive association between *T*. *vaginalis* serostatus and overall prostate cancer risk, and suggested the possibility of prostate carcinogenesis due to activation of oncogenes by IL-6-STAT3 signaling [5, 51]. Our recent study reported that IL-6 of prostate cells produced by *T*. *vaginalis* infection induces the proliferation of prostate epithelial cell as well as prostate cancer cells [31, 52]. Although *T*. *vaginalis* infection has been mentioned as a candidate for carcinogenesis of prostate cancers, *T*. *vaginalis* infection is still not accepted as a factor causing prostate cancer. Our previous studies showed that *T*. *vaginalis* induces an inflammatory response by producing inflammatory cytokines such as IL-1β, IL-6, CXCL8 and ROS in prostate cells, and by enhancing the migration of immune cells including monocytes, neutrophils and mast cells, and that inflammatory mediators released from prostate epithelial cells infected with *T*. *vaginalis* cause proliferation of stromal cells via crosstalk with mast cells [17, 19].

CCL2 and CXCL8 are chemokines that recruit monocytes and macrophages, as well as other immune cells, to sites of inflammation [53–55]. In this study, we showed that prostate epithelial cells stimulated by *T*. *vaginalis* produce cytokines such as IL-6, CCL2 and CXCL8, some of which induce the migration of THP-1 monocytes. Therefore, we suggested that *T*. *vaginalis* infection of prostate could create an inflammatory microenvironment by recruiting other immune cells and producing inflammatory cytokines.

Tumor-associated macrophages (TAMs) are the predominant immune cells in the tumor microenvironment [13]. Macrophages are generally divided into two types: M1, characterized by high level expression of IL-12, CXCL10, TNF-α, and inducible nitric oxide synthase (iNOS), and M2 by strong surface expression of macrophage mannose receptors (MMR, also known as CD206), CD163 and CD36, and high levels of TGF-β and IL-10 [25, 56]. TAMs are mainly composed of M2-type macrophages, and play crucial roles in the survival, proliferation and metastasis of cancer cells, and high TAM density generally correlates with a poor prognosis [24]. M2 polarization of macrophages is provoked by Th2 cytokines such as IL-4 and IL-13, whereas M1 polarization is stimulated by LPS and Th1 cytokines such as IFN-γ [25].

IL-6, a classical proinflammatory cytokine, promotes the formation to M2-type macrophages from TAMs, and is often viewed as a Th2 cytokine [26, 27]. IL-6 signaling is linked to activation of JAK and STAT3 and induces anti-inflammatory effects by promoting M2 macrophage polarization. Our findings indicate that the IL-6-containing conditioned medium of prostate epithelial cells co-cultured with *T*. *vaginalis* promotes M2 polarization of macrophages by upregulating M2 markers such as IL-10, TGF-β, CD36, CD206, and arginase-1, and that M2 polarization of THP-1-derived macrophages is regulated by the IL-6R/JAK signaling pathway. In addition, proliferation is a signature of M2 macrophages, which can accumulate in conditions where recruitment of monocytes is excluded, and IL-6 signaling regulates this proliferation [27, 28]. In this study, proliferation of THP-1-derived macrophages was induced by IL-6 signaling pathway.

The *c-M*yc gene, a member of the myc family of proto-oncogenes, plays a role in the control of lymphocyte homeostasis and the survival of myeloid cells [57, 58]. It is induced in macrophages by activation of STAT3 during alternative activation, and represents a new marker of the M2 phenotype [59, 60]. Cyclin D has been identified as a CSF-1-responsive gene that is the primary regulator of mononuclear cell survival, proliferation, and differentiation [61]. The response to M-CSF involves transcription of transient gene clusters in which cell cycle genes such as Cyclin D1 are overrepresented [25, 62]. Members of the Bcl-2 family are major regulators of apoptosis in mammalian cells, and expression of anti-apoptotic Bcl-2 protein induces production of the Th2 cytokine IL-13 via the JAK2/STAT3 signaling pathway [63]. M2-like macrophages induced by IL-6 and CCL2 were reported to induce cell survival, with upregulation of Bcl-2 protein [64]. Proliferating cell nuclear antigen (PCNA), an auxiliary protein of DNA polymerase and highly expressed during the cell cycle, is expressed in proliferating macrophages within populations of TAMs and is a proliferation-associated marker of human lymphoma-associated macrophages [65]. We showed above that cyclin D1 and PCNA expression in macrophages was enhanced by conditioned medium of prostate epithelial cells stimulated with *T*. *vaginalis* and decreased by blockade of IL-6R and JAK signaling. Thus, we suggest that IL-6 may induce proliferation of M2 macrophages by activating proliferation-related molecules through the IL-6R/JAK/STAT3 signaling pathway. Moreover, M2 macrophages promote angiogenesis, tumor growth, and metastasis [34], and their infiltration into the prostate tissue correlated with the aggressiveness of the cancers in 93 patients [40]. In this study, the conditioned medium of M2-like macrophages (M-TCM) increased the proliferation and migration of human prostate cancer cells (PC3, DU145 and LNCaP). The increase in proliferation and migration in TCM-stimulated prostate cancer cells is due to the effects of inflammatory cytokines such as IL-6 and CXCL8 in TCM, which is consistent with our previous study [31]. However, the conditioned medium (gp130+M-TCM), which inhibited M2 polarization of THP-1-derived macrophages by the IL-6 receptor antibody, did not induce proliferation and migration of prostate cancer cells. Therefore, these results suggest that M2 macrophages differentiated by TCM induce proliferation and migration of prostate cancer cells. In addition, PC3 and DU145 cells as androgen-independent cells are known to have stronger metastatic potentials than androgen-dependent LNCaP cells [66]. As a result of Fig 10, PC3 and Du145 showed higher migratory properties than LNCaP. It is expected that difference of the metastatic potentials among three prostate cancer cells may influence on the migration ability.

In conclusion, *T*. *vaginalis* stimulation of prostate epithelial cells induces an inflammatory response by producing cytokine such as IL-6 and chemokines such as CCL2 and CXCL8, and these chemokines promote the migration of THP-1 monocytes. IL-6 induces the proliferation and M2-type polarization of macrophages through the IL-6R/JAK signaling pathway. Finally, interaction between THP-1-derived macrophages and inflammation due to *T*. *vaginalis* infection enhances the proliferation and migratory ability of prostate cancer cells. Taken together, our findings suggest that *T*. *vaginalis* may have an important effect on the tumor microenvironment, promoting progression of prostate cancers by inducting M2 macrophage polarization. However, further studies are needed to elucidate the distinct mechanism between *T*. *vaginalis* infection and prostate cancer.

## Supporting information

S1 Table PCR primer sequences. The sequences of all primer pairs were acquired from the international nucleotide sequence database and designed using Primer3 software.
(DOC)

S1 Fig Involvement of the IL-6 signaling pathway in M2-type macrophage polarization of THP-1 cells. THP-1 cells were treated with 100 nM PMA for 24 hr, and cultured with TCM for 72 hr. To examine involvement of IL-6 signaling in M2 macrophage polarization, M0 macrophages were pretreated with anti-gp-130 (IL-6 receptor beta, 100 ng/ml) or anti-IL6 receptor alpha antibody (100 ng/ml) before addition of TCM. Macrophages were incubated with ruxolitinib alone (JAK inhibitor, 10 μM) to determine the cytotoxicity of ruxolitinib or with anti-IgG isotype antibody (100ng/ml) before adding TCM to determine the non-specific antibody binding. **(A)** Production of IL-10 and TGF-β as M2 macrophage markers and IL-12 and CXCL-10 as M1 macrophage markers was measured by ELISA assays. **(B)** Proliferation of macrophages was evaluated by CCK-8 assays. **(C)** Expression of p-STAT3 and cyclin D1 protein was determined by western blot. Graph represent densitometric analysis (means of three independent western blot experiments). Data are means ± SD of three independent experiments. *p<0.05 versus THP-1-derived macrophage (M0). #*p*<0.05 versus conditioned medium of RWPE-1 stimulated with *T*. *vaginalis* (TCM). NS = not statistically significant, TCM: conditioned medium of RWPE-1 stimulated with *T*. *vaginalis*, Rux: Ruxolitinib.
(TIF)
