# Supplementary figures and images for: IL-6 produced by prostate epithelial cells stimulated with Trichomonas vaginalis promotes proliferation of prostate cancer cells by inducing M2 polarization of THP-1-derived macrophages
Source: PLoS Negl Trop Dis. 2020 Mar 20;14(3):e0008126. doi: 10.1371/journal.pntd.0008126 (PMC7138318; doi:10.1371/journal.pntd.0008126)

**Supplementary Table 1. PCR primer sequences.**


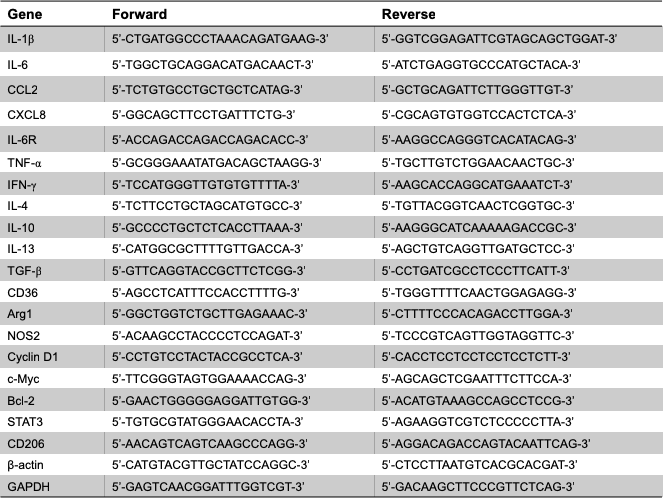

Supplement: S1 Table — The sequences of all primer pairs were acquired from the international nucleotide sequence database and designed using Primer3 software. (DOC) [file pntd.0008126.s001.doc]

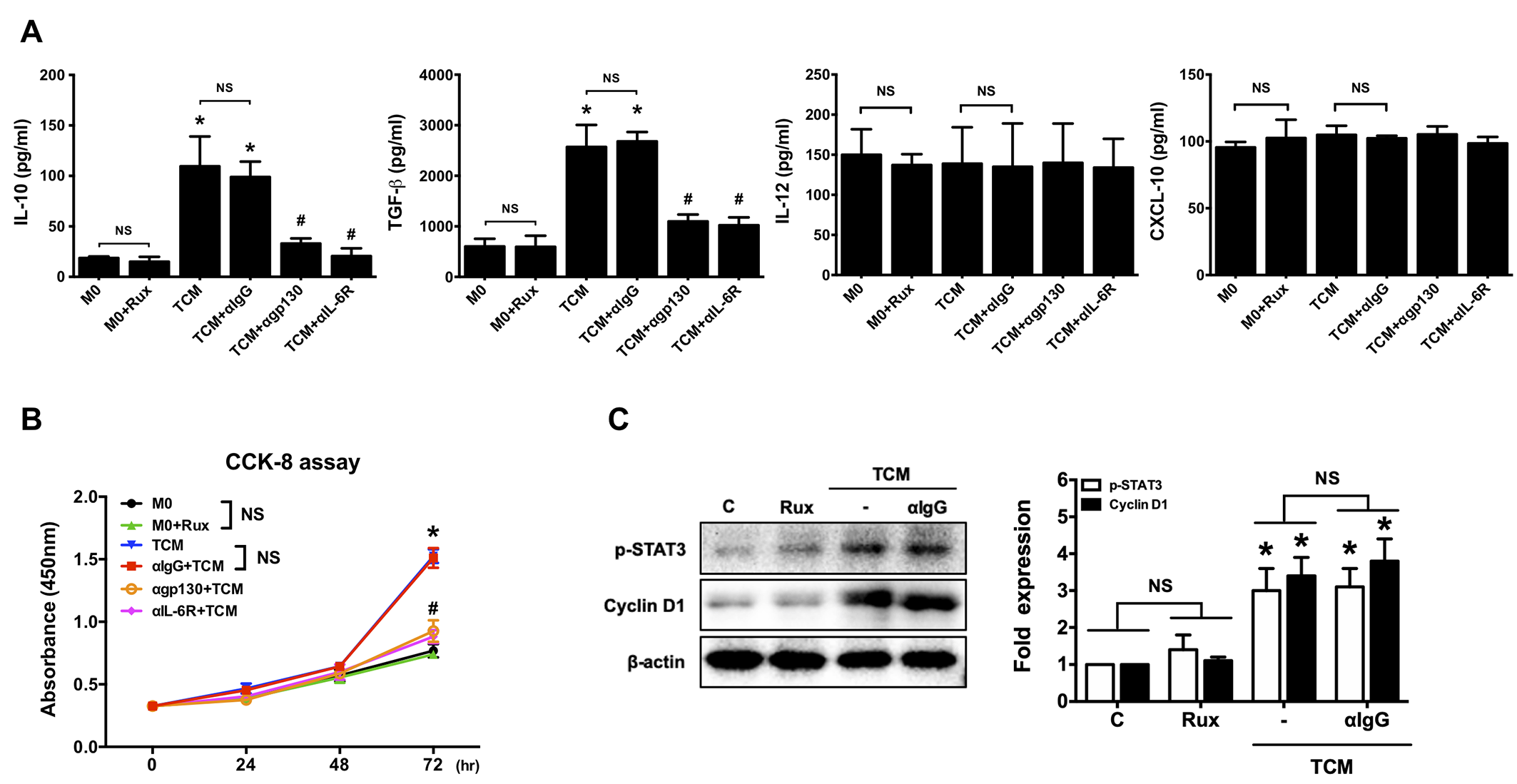

Supplement: S1 Fig — THP-1 cells were treated with 100 nM PMA for 24 hr, and cultured with TCM for 72 hr. To examine involvement of IL-6 signaling in M2 macrophage polarization, M0 macrophages were pretreated with anti-gp-130 (IL-6 receptor beta, 100 ng/ml) or anti-IL6 receptor alpha antibody (100 ng/ml) before addition of TCM. Macrophages were incubated with ruxolitinib alone (JAK inhibitor, 10 μM) to determine the cytotoxicity of ruxolitinib or with anti-IgG isotype antibody (100ng/ml) before adding TCM to determine the non-specific antibody binding. (A) Production of IL-10 and TGF-β as M2 macrophage markers and IL-12 and CXCL-10 as M1 macrophage markers was measured by ELISA assays. (B) Proliferation of macrophages was evaluated by CCK-8 assays. (C) Expression of p-STAT3 and cyclin D1 protein was determined by western blot. Graph represent densitometric analysis (means of three independent western blot experiments). Data are means ± SD of three independent experiments. *p<0.05 versus THP-1-derived macrophage (M0). #p<0.05 versus conditioned medium of RWPE-1 stimulated with T. vaginalis (TCM). NS = not statistically significant, TCM: conditioned medium of RWPE-1 stimulated with T. vaginalis, Rux: Ruxolitinib. (TIF) [file pntd.0008126.s002.tif]
